# Mitochondrial Dysfunction as a Convergent Mechanism of α-Synuclein Toxicity: A Systematic Review of In Vitro Fibrillar Models

**DOI:** 10.1007/s12035-026-06287-9

**Published:** 2026-10-28

**Authors:** Hanadi Al Tbeishat, Douglas G Ward, Ilse S Pienaar, Richard J Elsworthy

**Affiliations:** 1 Department of Biomedical Sciences, College of Medicine and Health, University of Birmingham, Birmingham, B15 2TT UK; 2 Department of Cancer and Genomic Sciences, College of Medicine and Health, University of Birmingham, Birmingham, B15 2TT UK; 3 School of Sport, Exercise and Rehabilitation Sciences, College of Life and Environmental Sciences, University of Birmingham, Birmingham, B15 2TT UK; 4 Centre for Human Metabolomics, North-West University, Potchefstroom, South Africa

**Keywords:** α-Synuclein, Mitochondrial dysfunction, Synucleinopathies, Oxidative stress, Neurodegeneration, Parkinson’s disease

## Abstract

Synucleinopathies, including Parkinson’s disease, Dementia with Lewy bodies, and Multiple System Atrophy, are characterised by intracellular accumulation of misfolded α-synuclein (αSyn). Although αSyn aggregation is central to disease pathogenesis, the mechanisms linking αSyn to progressive neurodegeneration remain incompletely understood. Mitochondrial dysfunction is recognised as a key pathological process, particularly in experimental models using exogenously applied αSyn species, including preformed fibrils (PFFs). This systematic review synthesises evidence from mammalian in vitro studies that examined the effects of disease-associated αSyn conformations, including monomers, oligomers and PFFs, on mitochondrial structure and function. Following PRISMA 2020 guidelines, EMBASE, MEDLINE, and PsycInfo were searched to identify eligible peer-reviewed studies published up to March 2026, yielding 33 included studies. Models encompassed neuronal, astrocytic, microglial, endothelial, fibroblast, and induced pluripotent stem cell-derived systems, enabling comparison across cell types, αSyn conformations, and genetic backgrounds. Across included studies, oligomeric and fibrillar αSyn consistently induced mitochondrial depolarisation, impaired oxidative phosphorylation, reduced ATP production, complex-I dysfunction and increased reactive oxygen species generation. Additional mitochondrial pathological changes included fragmentation, cristae disruption, impaired TOM20-mediated protein import, calcium dysregulation, defective mitophagy and increased heteroplasmy i.e. accumulation of damaged mitochondria. These alterations frequently preceded cell death and were amplified in dopaminergic and genetically vulnerable models, including αSyn A53T, SNCA triplication, and PARK2-associated transgenic systems. Our review highlights mitochondrial dysfunction as a mechanism underlying αSyn toxicity, whilst suggesting methodological heterogeneity can limit cross-study translation. Priorities for future research using ‘in vitro’ models of PFF synucleinopathies include standardised fibril preparation protocols, improved mitochondrial phenotyping, and use of human multicellular and organoid models.

## Introduction

Synucleinopathies, including Parkinson’s disease (PD), Parkinson’s Disease Dementia (PDD), Dementia with Lewy bodies (DLB), and Multiple System Atrophy (MSA), are progressive neurodegenerative diseases characterised by pathological intracellular accumulation of aggregated, misfolded alpha-synuclein (αSyn) protein [1, 2]. In PD, PDD and DLB, αSyn pathology is classically observed as Lewy bodies (LBs) and Lewy neurites, respectively within disease-vulnerable neuronal somas or neurite processes, while MSA is distinguished by αSyn-positive glial cytoplasmic inclusions, particularly occurring within oligodendrocytes [1–3]. Despite distinct clinical presentations and anatomical distributions of pathology, these diseases are united by a common pathological hallmark: the conversion of soluble physiological αSyn into aggregated species associated with neurodegeneration.

PD is the most common synucleinopathy and the second most prevalent neurodegenerative disease after Alzheimer’s disease, affecting millions of people worldwide [4]. In addition, with progressive ageing being the single greatest risk factor for developing PD, the prevalence of PD is expected to rise as populations age, making it a global health challenge. The clinical presentation of PD is defined by motor symptoms which include bradykinesia, rigidity, tremor and postural instability, but non-motor features such as cognitive decline, autonomic dysfunction, sleep disturbance and psychiatric symptoms are increasingly recognised as major contributors to disease burden [5, 6]. Whilst motor-related symptoms reflect degeneration of dopaminergic neurons in the Substantia Nigra pars compacta (SNpc), cognitive and psychiatric manifestations of PD represent broader disruption of non-dopaminergic neuronal networks, especially ones relying on acetylcholine-related transmission [7, 8]. Although the aetiology of PD remains incompletely understood, with no single causative factor identified, the discovery of mutations in the *SNCA* gene for encoding αSyn, in familial PD, together with the identification of misformed αSyn as the principal component of LBs, has established αSyn as a central mediator of synucleinopathy pathogenesis and related disease progression [3, 9]. In a key study by Prusiner and colleagues, it was experimentally demonstrated that human αSyn aggregates possess authentic self-propagating prion-like biochemical behaviour [10]. The study further strengthened the hypothesis that neurodegenerative disease can result from templated protein misfolding, whilst also introducing the idea that different conformational strains of αSyn may explain the clinical and pathological diversity of synucleinopathies [10]. However, the precise set of mechanisms through which pathological αSyn species drive progressive cellular dysfunction and neurodegeneration remain incompletely understood.

Under physiological conditions, αSyn is a 140-amino-acid protein enriched at presynaptic terminals, where it contributes to synaptic vesicle trafficking, neurotransmitter release and stabilisation of cell membranes [11–13]. αSyn exists in equilibrium between soluble cytosolic and membrane-bound states, exhibiting high affinity for lipid-rich membranes [14, 15]. Importantly, αSyn has also been implicated in mitochondrial bioenergetics, mitochondrial quality control, and in mediating communication between the endoplasmic reticulum and mitochondria at mitochondria-associated membranes (MAMs) [16, 17]. Given this, disruption in physiological αSyn signaling holds potential to significantly alter cellular metabolic function. In this regard, αSyn can undergo conformational changes from predominantly α-helical states toward β-sheet-rich oligomers or fibrillar assemblies during progressive synucleinopathy [18, 19]. Soluble oligomers, protofibrils and mature fibrils differ in their structure, membrane interactions, seeding capacity and toxicity, making the relationship between αSyn aggregation and cellular injury a central question in synucleinopathy research [20, 21]. Once in an aggregated form, αSyn has been proposed to propagate through prion-like mechanisms, with new αSyn aggregates acting as nascent seeds in neighboring cells. Specifically, in PD, fibrillar αSyn species are proposedly released from affected LB-harboring cells through exocytosis, vesicular release or leakage from degenerating neurons, before being taken up by neighboring cells through endocytic and receptor-mediated mechanisms [22–24]. Once internalised, aggregated αSyn can seed the conversion of endogenous soluble αSyn into insoluble, phosphorylated and ubiquitinated inclusions, thereby amplifying pathology within vulnerable cellular networks [25, 26]. This not only provides a mechanistic framework for understanding synucleinopathy progression but also highlights the need for experimental models that can recapitulate such processes, and, at a molecular-cellular resolution, isolate the cellular and metabolic effects of αSyn seeding and related propagation.

Preformed fibril (PFF) experimental models have become frequently used for studying mechanisms of αSyn-related toxicity. In such models, fibrillar αSyn is exogenously applied to create cell culture models and intracerebrally delivered to produce animal models of synucleinopathy-related disease [25–28]. These models are deemed highly valuable for studying intracellular processes following acute exposure to specific αSyn conformations and for testing novel or repurposed therapeutics for the ability to diminish αSyn fibril spread and/or level. PFF exposure can reproduce several disease-relevant features, including αSyn phosphorylation at serine–129 which is a major post-translation modification in synucleinopathy, aggregation into larger insoluble fibrils, impaired proteostasis, synaptic dysfunction, activation of inflammatory cascades, with eventual cell death [25, 26, 29–31]. However, interpretation of in vitro fibrillar models is complicated by substantial methodological heterogeneity in terms of production, quality control workflows and delivery. Specifically, the biological effects of exogenously applied αSyn depend on the conformation of the species used, including whether cells are exposed to monomers, oligomers, protofibrils, sonicated PFFs or mature fibrils [20, 29, 30]. Moreover, differences in PFF processing protocols, exposure durations, and treatment concentrations can alter seeding efficiency and downstream cellular responses in PFF models. These variables underscore the need for rigorous characterisation of fibril preparations to ensure reproducibility.

Although the precise toxic synergistic relationship between aggregated αSyn and mitochondria remains incompletely understood, mitochondrial dysfunction is one of the most consistently implicated cellular consequences of exogenous application of αSyn. Neurons are highly dependent on efficient mitochondrial function for adequate production of ATP, calcium buffering, redox homeostasis, synaptic activity and apoptotic regulation. This dependency renders mitochondria particularly susceptible to PFF-induced toxicity, shown to affect these mitochondria-related functions [32, 33]. Mitochondria undergo active transport along neuronal axons to reach distal compartments i.e.synapses, where they provide ATP to meet localized energy requirements. [34]. Thus, a loss of mitochondrial function can contribute to synaptic dysfunction and, ultimately, to neuronal death [7, 34].

αSyn has been implicated in mitochondrial dysfunction through several interconnected mechanisms. Aggregated αSyn can interact with mitochondrial membranes and disrupt the function of key proteins involved in oxidative phosphorylation, including respiratory complex I, ATP synthase, voltage-dependent anion channels, and TOM20-mediated protein import pathways [35–37]. These disruptions can impair electron transport along the mitochondrial proton gradient, decrease ATP production, and increase reactive oxygen species (ROS) generation thereby promoting oxidative stress [33, 38, 39]. In addition, αSyn aggregation can disturb mitochondrial calcium homeostasis and impair mitophagy, creating a self-amplifying cycle of oxidative and proteostatic stress that further exacerbates mitochondrial dysfunction [15, 40, 41].

With growing experimental evidence from in vitro* models* of αSyn toxicity impacting mitochondrial function, this review aims to synthesis existing literature*.* Specifically, this review evaluates how αSyn species, cell types and treatment conditions influence mitochondrial outcomes, and considers whether mitochondrial dysfunction represents a consistent mechanism that links αSyn aggregation to cellular toxicity. Ultimately, our review identifies the mitochondrial functional pathways most consistently associated with αSyn toxicity, highlighting key mechanisms of disease pathogenesis for guiding the development of future experimental models for PD and related synucleinopathies. With therapeutic targeting of mitochondria and pathological αSyn emerging as a viable treatment approach to potentially slow the progression of synucleinopathies such as PD [35], the present review offers a timely critical reflection on accumulating studies reporting on αSyn-mitochondria synergism as a driver of synucleinopathies’ onset and progression.

## Methods

### Study Selection

A literature search was conducted on 12 March 2026 using the Ovid database platform [36] (https://ovidsp.ovid.com/), which includes Medline, EMBASE and the American Psychological Associations’ PsycInfo. The search strategy followed the PRISMA guidelines [37] to ensure transparency and consistency in systematic review reporting. Truncation was denoted by an asterisk, aimed at capturing multiple word variants. Search keywords (excluding Boolean operators) were as follows: (((“synucleinopath*” OR “alpha-synuclein” OR “Parkinson’s disease*” OR “Lewy body 2adj (dementia* OR disease*)” OR “multiple system atrophy”) AND (“cellular model” OR “cell model” OR “in vitro” OR “neuronal model” OR “primary neuron*” OR “induced pluripotent stem cell*” OR “iPSC-derived neuron” OR “neuroblastoma*”) AND (“subcellular mechanism*” OR “mitochondrial dysfunction” OR “autophagy” OR “protein aggregation” OR “neuronal degeneration” OR “oxidative stress” OR “lysosomal dysfunction” OR “cell viability” OR “membrane”)) NOT (“clinical*” or “animal model” or “review”)). Use of the term 2adj directed the search engine to find a term appearing within exactly 2 words of the words placed within the parentheses. Furthermore, search terms were combined using Boolean operators and database-specific field tags (e.g. mp. for multi-purpose searching), enabling retrieval of records containing the specified terms across titles, abstracts, subject headings, and keyword fields. The mp. search terms were tailored to each database, incorporating appropriate medical subject headings (MeSH) and the Ovid ‘explode’ function was used where relevant to capture broader topic coverage. To maximize retrieval and reduce risk of omission, mapped keywords were also searched independently. Additional exclusions were for (1) conference abstracts and (2) studies published outside the publication focus window period, namely between 1981 and March 2026.

### Inclusion Criteria

1. Peer-reviewed experimental studies
2. Studies using in vitro model systems
3. Studies applying exogenous αSyn monomers, oligomers or PFFs
4. Studies that report on structural or functional changes related to mitochondrial dysfunction linked to αSyn pathology
5. Publications in English
6. Studies published between March 1981 and March 2026

### Exclusion Criteria

1. Abstracts, editorials, conference materials, reviews, meta-analyses, or papers lacking full experimental data
2. Studies that made use of in vivo models
3. Research unrelated to synucleinopathies or αSyn pathology
4. Absence of a link between αSyn aggregation and mitochondrial dysfunction
5. Studies not introducing αSyn exogenously
6. Use of non-mammalian species
7. Studies involving clinical or human subjects only, without use of model systems
8. Non-English language publications

### Data Extraction

Information was extracted by two authors (HaT, RJE) using a structured three-stage exclusion process to identify studies for full review. During phase 1 (study identification), duplicate entries, posters, and conference abstracts were removed by HaT. Phase 2 (study screen) involved assessing titles and abstracts against the predefined inclusion and exclusion criteria to filter out irrelevant records; this was carried out independently by HaT and RJE. For phase 3 (study eligibility), full-text articles were thoroughly reviewed by both HaT and RJE, with detailed information that was collected from them with regard to the conformational status of αSyn used, cellular model(s) utilised, study type, key findings, and mitochondria-related outcomes to determine suitability for inclusion in the systematic review (Table 1).

**Table 1:** Summary of published studies included as part of the current systematic review, which investigated the effects of αSyn exposure on mitochondrial outcomes

Ref	Model/cell line	αSyn conformation, -dosage and -exposure duration	Mitochondrial function measures	Key findings
[39]	HEK293 & SH-SY5Y cells	WT recombinant human fibrils; 5 μg; >48 h	• MMP • Oxidative stress • Lipid peroxidation • Mitochondrial-cytoskeletal interaction	Cofilin-1 activation and mt translocation cause ΔΨm depolarization, ↑ROS, lipid peroxidation and apoptosis via pro-apoptotic factor release
[47]	SH-SY5Y cells	WT recombinant human oligomers; 0.3 µM; 24 h	• Bioenergetics	↓Basal and maximal mt respiration impair bioenergetics
[48]	hiPSC-derived microglia	WT recombinant human PFF; 0.5 μM; 24 h	• Redox imbalance and oxidative stress • MMP	Mt dysfunction marked by glycolytic shift, ↓mitochondrial content, ↑oxidative stress (iNOS and SOD2) and ΔΨm collapse in microglia
[49]	SH-SY5Y cells	WT recombinant human PFF; 1 µM; 48 h	• MMP • Oxidative stress • Lipid peroxidation • Mitochondrial morphology	Increased ΔΨm depolarization, METC complex I inhibition, mt damage (swelling and cristae disruption), ↑ROS, MDA and H_2_O_2_ elevation, ↓antioxidants (SOD and GSH), culminating in apoptosis
[69]	hiPSC-derived cortical neurons	S129A-mutant recombinant human PFFs; 10 µg/ml; 3, 6, 13 dpt	• Mitochondrial motility	↓Mt motility without changes in mt number
[40]	Rat primary midbrain astrocytes, hiPSC-derived astrocytes and the U87 cell line	WT and A30P/A53T double-mutant recombinant human aggregates; 0.5 µM; 24–48 h	• MMP	Delayed ΔΨm depolarization following ER stress and calcium dysregulation in astrocytes
[41]	hCMEC/D3 cells	WT recombinant human oligomers; 10% v/v; 12 h	• Oxidative stress	↓ΔΨm, NO-mediated impairment and matrix metalloproteinase activation that disrupted the BBB
[50]	3D ex vivo organotypic hippocampal slice cultures	Recombinant human PFFs and monomers; 2.5 μM αSyn monomers and/or 500 nM αSyn PFF; 57 dpt	• Mitochondrial dynamics • Mitochondrial structure • Mitophagy	↓ATP, mt cristae loss, mt compression, impaired autophagy and mt sequestration in autophagosomes
[64]	SH-SY5Y cells	αSyn oligomers; 25 μmol/L; 24 h	• Oxidative stress	↑ROS contributing to oxidative stress via miRNA and regulatory pathways
[65]	Primary rat mesencephalic culture: ~70% GABAergic neurons; 8% dopaminergic	WT recombinant human oligomers; 250 nmol/L; 168 h	• Oxidative stress • Apoptosis • Lysosomal-mt crosstalk	Progressive mt dysfunction with ↑ROS, CytC/AIF release, caspase activation and lysosomal-autophagic stress
[51]	Primary mouse cortical, mesencephalic and enteric neuronal cell cultures: 2–4% dopaminergic neurons	WT recombinant human oligomers; 2 µM or 0.2 µM, then 1 µM or 0.1 µM (after 2 days)	• MMP • Bioenergetics	ΔΨm depolarisation of dopaminergic neurons with effects proportional to uptake and retrograde transport; Endogenous αSyn supports mt respiration and glycolysis αSyn KO neurons had impaired bioenergetics
[52]	Primary rat cortical co-cultures: hiPSC-derived neurons	αSyn WT, αSyn mutants (A53T, A30P and E46K) and human recombinant monomers and oligomers; 500 nM; n.s	• Oxidative stress • MMP • Apoptosis	METC complex I inhibition, NADH oxidation impairment, Δψm disruption, ↓ATP, ↑ROS, mPTP opening and caspase-dependent apoptosis; Energy metabolism becomes dependent on ATP hydrolysis via METC complex V
[43]	hiPSC-derived microglia, hiPSC-derived A9-dopaminergic neurons	WT recombinant human αSyn fibrils; 1 µM; 6 h	• Oxidative stress • MMP • Neuroinflammation • Mitochondrial structure	↑ROS, ↓ΔΨm, mtDNA release triggers inflammasome activation
[42]	Primary mouse microglial cells	WT recombinant human and mouse αSyn fibrils; 1 μM; 6, 12, 18, and 24 h	• Oxidative stress • MMP • Neuroinflammation	↑ROS, ↓ΔΨm loss and microglial activation
[44]	hiPSC-derived astrocytes	WT recombinant human αSyn (monomers, PFFs, or F110 variant [αSyn (1–110) fibrils]); 10 μM; 6 days	• Bioenergetics	Impaired mt respiration with ↓oxygen consumption (↓basal, uncoupled and ATP-linked oxygen consumption) in hiPSC-astrocytes; mt mass unchanged
[53]	SH-SY5Y cells	WT recombinant human αSyn monomer/oligomer mix; 10 μM; 24 or 48 h	• MMP • Oxidative stress • Energy metabolism • Mitophagy	ΔΨm depolarization, ↑ROS, ↓ATP, impaired mitophagy via P2X7 receptor activation, culminating in energy metabolism disruption and apoptosis
[70]	SH-SY5Y cells	PMCA-generated WT recombinant human fibrils; 5% v/v; 72 h	• Bioenergetics	Hyperactive mt respiration without ETC impairment or glycolysis alteration
[54]	P1 mouse or rat cortical neurons	WT recombinant human & mouse monomer PFF; 140 nM; 8–9 days	• Bioenergetics • Mitochondria-fibril co-localisation	PFFs reduce mt basal/maximal respiration and mt respiratory resilience
[67]	Primary mouse cortical/hippocampal neurons	WT recombinant human monomer/PFFs; 2 μg/ml (PFF); 4 μg/ml (monomers); 5–6 days	• Mitochondrial structural damage • Mitophagy	Mt fragmentation, -depolarization, -metabolic stress, mitophagy, tau phosphorylation and MAPK pathway activation
[55]	Primary co-cultures of rat neurons and astrocytes; iPSC-derived neurons (SNCA triplication)	WT human recombinant monomers/oligomers; Monomeric αSyn 100 nM; Oligomeric αSyn (~1% oligomers in mixture) 100 nM monomers (~1 nM oligomers); n.s	• MMP • Oxidative stress • Lipid peroxidation • Apoptosis	METC complex I impairment, ↑ROS, ΔΨm depolarization, PTP opening, mitochondrial swelling, oxidation of ATP synthase β subunit and mt lipid peroxidation, altered NADH redox state and neuronal death
[61]	SH-SY5Y cells	WT & A53T human recombinant monomers or oligomers; 10 μM; 24 and 48 h	• Apoptosis • Oxidative/nitrosative stress	Mt membrane disruption, NO release, and S-nitrosylation impair mitochondrial quality control and promote apoptosis
[62]	Primary mouse microglial cells; the mouse microglia BV2 cell line	WT recombinant human PFFs; 200 nM; overnight	• Oxidative stress	NOX2 activation and oxidative stress via integrin CD11b and Rho signaling
[45]	Primary mouse hippocampal neurons	WT recombinant human monomer and PFFs; 2 or 4 μg/mL; 3.5 days	• MMP • Apoptosis • Mitophagy	ΔΨm depolarization, CytC accumulation and release, ↑mt fragmentation, impaired mt protein import and phosphorylated ACC1 sequestration indicating metabolic stress. Localization to MAMs and presence in Parkin-positive vacuoles indicate activated mitophagy
[56]	SH-SY5Y cells	WT Recombinant human monomer/PFF; 10 μM; 24 h	• Energy metabolism	Disrupted mt calcium homeostasis via direct activation of P2X7 receptors, leading to intracellular calcium overload, extracellular ↑ATP and ↓ATP degradation
[58]	SH-SY5Y cells HEK293 cells	Monomers, oligomers, dopamine-modified, nitrated, S129E mutant αSyn; 200 nM; 24 h	• ΔΨm • Oxidative stress • Bioenergetics • Mitochondrial protein import	Impaired mt protein import via disrupted TOM20-TOM22 interactions, leading to ↓ mt respiration and ΔΨm, and ↑ROS
[21]	hiPSC-derived cortical neurons (WT and 3× SNCA); Primary rat midbrain neurons	WT human recombinant monomeric, oligomeric (compact, β-sheet-rich, stable) αSyn; 10 pM-40 nM (mainly 40 nM); Acute	• Oxidative stress	Oligomers induce ↑ cytosolic and mt ROS, glutathione depletion, lipid peroxidation and neuronal toxicity
[63]	Primary mouse mesencephalic dopaminergic neurons	WT recombinant human PFFs; 2.5 μg/ml; 2 weeks	• Oxidative stress	Increased mt oxidative stress in dopaminergic dendrites, driven by ↑calcium and NADPH oxidase
[59]	Primary human cortical neurons; Primary human astrocytes; Human skin-derived fibroblasts	WT recombinant human αSyn oligomers; 5 μM; 24 h	• Bioenergetics • Mitochondria-oligomer co-localisation	Direct αSyn localization to mt that associated with ↓Oxygen consumption, ↑basal control ratios, ↓uncoupled respiration indicating impaired mt capacity
[66]	SH-SY5Y cells	WT and E46K αSyn mutant; recombinant human oligomers; 5 μM; 4 h, 24 h, 72 h	• Oxidative stress	Time-dependent ↓mt activity and ↑ROS. The E46K αSyn mutant induced greater mt impairment than WT αSyn
[68]	Differentiated human LUHMES dopaminergic neuronal cells	WT recombinant human PFF; 1 μg/mL; 48 h	• Oxidative stress • Mitochondrial protein QC	Disruption of protein QC, including mt-associated chaperones e.g. Hsp60; Energy production (i.e. ATP levels) maintained but dependented on compensatory mechanisms
[46]	Primary mouse cortical neurons	WT recombinant human PFF; n.s.; 5–14 days	• MMP • Oxidative stress	αSyn binds ClpP via its NAC domain, inhibiting its proteolytic activity and disrupting mt proteostasis. This led to accumulation of misfolded proteins within mt, ↑mt ROS, ↓ΔΨm and ATP, collectively indicating a collapse in mt energy metabolism

Abbreviations used: *ACC1* Acetyl-CoA carboxylase 1, *Agg* Aggregation, *AIF* Apoptosis-inducing factor, *BBB* Blood–brain barrier, *CD11b* Cluster of Differentiation 11 b, *ClpP* Caseinolytic protease P, *CytC* Cytochrome C, *DIV* day in vitro, *Dpt* day post treatment, *ER* endoplasmic reticulum, *GSH* glutathione, *h* hours, *hiPSC* human induced pluripotent stem cells, H_2_O_2_ hydrogen peroxide, *iNOS* Inducible nitric oxide synthase, *KO* knock-out, *MDA* malondialdehyde, *Mt* mitochondria, *MAMs* mitochondrial associated membranes, *mtDNA* mitochondrial DNA, *METC* mitochondrial electron transport chain, *MMP/ΔΨm* mitochondrial membrane potential, *mPTP* mitochondrial permeability transition pore, *MAPK* misfolding cyclic mitogen-activated protein kinase, *NAC* non-amyloid-β component, *NOX* NADPH oxidase, *NO* nitric oxide, *ns* not stated, *PMCA* plasma membrane Ca^2^⁺-ATPase, *PFFs* preformed fibrils, *QC* quality control, *Rho* Ras homolog, *ROS* reactive oxygen species, *Ref* reference, *SOD* superoxide dismutase, *WT* wild-type

## Results

### Study Selection

**Fig. 1:**
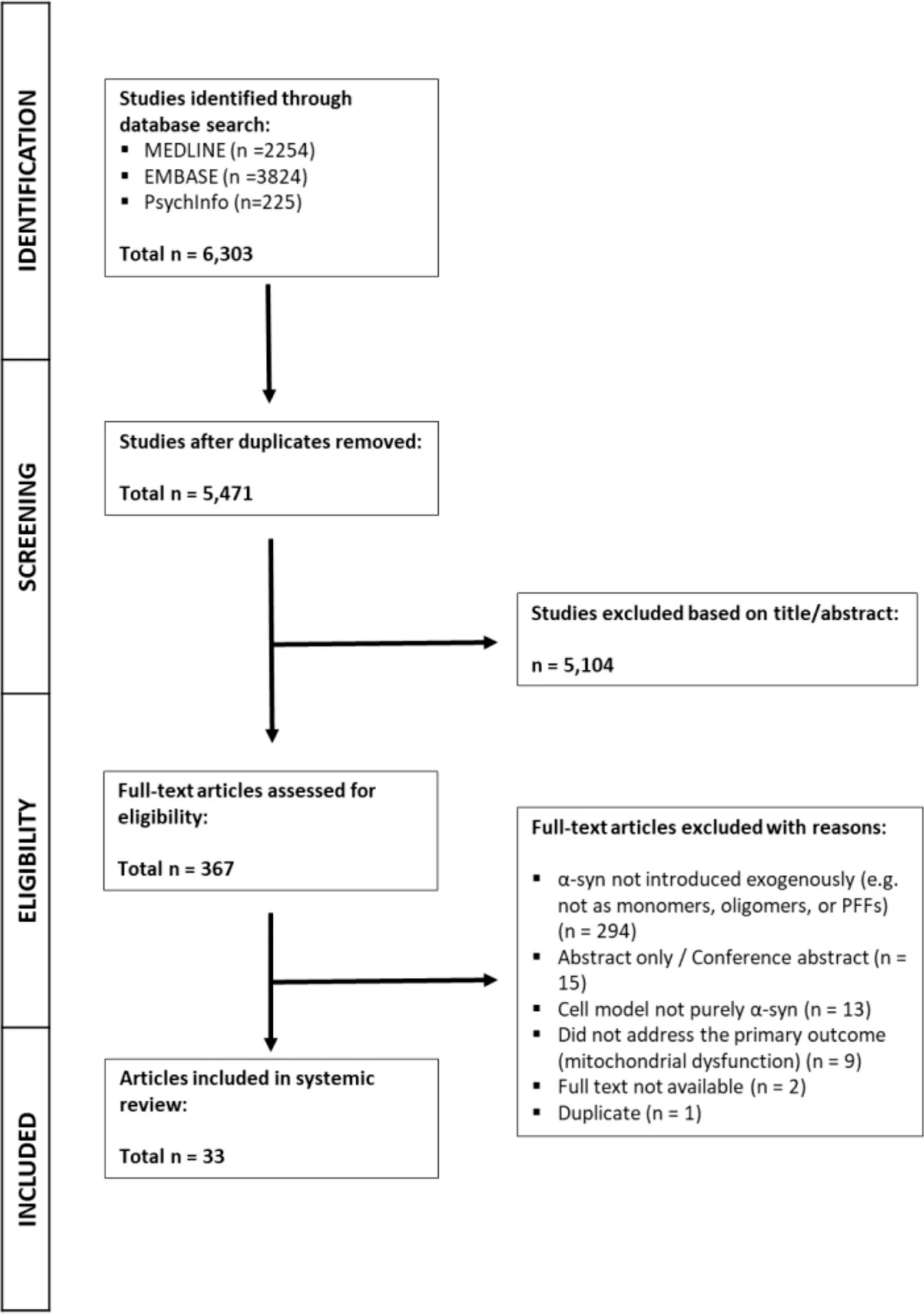
PRISMA flow diagram depicting the process of study identification, screening, eligibility assessment, and final inclusion for the systematic review

The database search yielded 6303 records (Medline = 2254, EMBASE = 3824, PsycInfo = 225). After removing duplicate titles, the abstracts of 5471 records were screened, of which 5104 were excluded. A total of 367 full-text articles were then assessed for eligibility, and 33 studies met the predefined inclusion criteria. The study selection process is shown in Fig. 1.

### Characteristics of Included Studies

A total of 33 peer-reviewed experimental studies published between March 2013 and March 2026 were included. This was expected since the use of PFF was introduced in the field in early 2011 [25]. All investigations employed in vitro cell-based systems to examine the effects of exogenously applied αSyn—in its monomeric, oligomeric, or PFF formats—on various aspects of mitochondrial function. The models most frequently utilized included SH-SY5Y neuroblastoma cells and primary rodent neurons, while several studies extended their analyses to iPSC-derived neurons, astrocytes, microglia, endothelial cells, fibroblasts, and organotypic slice cultures. This diversity of models reflects an effort to capture cell-type-specific vulnerabilities to αSyn induced stress. The mitochondrial outcomes assessed encompassed membrane potential, ROS generation, respiration and ATP production, mitochondrial morphology and dynamics, and mitophagy. Collectively, these endpoints provided complementary insights into both functional and structural consequences of αSyn exposure. Several studies incorporated genetic backgrounds linked to PD to explore potential gene-environment susceptibility to αSyn-induced mitochondrial dysfunction (Table 1), whilst others reported metabolite-related alterations resulting from various αSyn treated cell models (Table 2).

**Table 2:** Summary of metabolite alterations reported in published work that utilised αSyn exogenously treated cell model systems

Ref	Model/cell line	αSyn conformation, -dosage and -exposure duration	Mitochondrial functional assay	Metabolite function	Altered metabolites	Key findings
[48]	hiPSC-derived microglia	WT human recombinant; 0.5 μM αSyn PFFs; 24 h	2-NBDG-based glucose uptake assay for flow cytometry	• Glycolysis	• Glucose uptake	Enhanced glycolysis (~3 × glucose uptake), indicating mitochondrial dysfunction and a pro-inflammatory phenotype
[50]	3D ex vivo organotypic hippocampal slice cultures	WT human recombinant 2.5 μM αSyn monomers and/or 500 nM αSyn PFF; 57 dpt	Mitochondrial ToxGlo™ assay	• Energy metabolism	• ATP	ATP significantly reduced in PFF- or combo-treated slices; monomer alone caused no ATP change
[52]	Primary rat cortical co-cultures; hiPSC-derived neurons;	WT and mutant (A53T, A30P and E46K) human recombinant αSyn oligomers; 500 nM; n.s	ATP bioluminescence assay; NADH autofluorescence imaging	• Energy metabolism	• ATP • NADH	Complex I inhibition induces NADH accumulation, reduced Δψm, and ATP depletion; A53T cells sustain Δψm through reverse ATP synthase activity, indicating impaired bioenergetics
[60]	SH-SY5Y cells	WT human recombinant 500 nM αSyn PFFs; 14 dpt	NAD-GloTM™ assay	• Energy metabolism	• NAD+	PARP-1 hyperactivation depletes NAD⁺ (~60%), increases PAR, promotes αSyn aggregation, DNA damage and metabolic stress
[53, 56]	SH-SY5Y cells	WT recombinant human monomer/oligomer mix; 10 μM; 24 h		• Energy metabolism	• ATP	Mitochondrial depolarization and reduced ATP via P2X7 activation; AMPK inhibition disrupts energy homeostasis
[55]	Primary co-cultures of rat neurons and astrocytes iPSC-derived neurons (SNCA triplication)	Mixture of WT human recombinant monomeric and oligomeric αSyn; (100 nM monomers, ~1 nM oligomers); 4–8 h	NADH autofluorescence imaging	• Energy metabolism	• NADH	Complex I dysfunction promotes mPTP-mediated NADH collapse, mitochondrial depolarization, ROS accumulation and bioenergetic failure
[57]	SH-SY5Y cells	WT human recombinant monomers, oligomers and/or PFFs; 5 μM; 24 h	NMR	• Lipid metabolism • Amino acid metabolism • Energy & oxidative stress • Neurotrans-mission	• PC • 3GPC • myo-inositol • butyric acid • 3-hydroxyvalerate • Glycine • Glutamine • l-alloisoleucine • Creatinine, • Taurine • Dimethylamine • ATP • ACh • Taurine	αSyn oligomers promote membrane remodeling (↑3GPC), while fibrils induce mitochondrial dysfunction (↓phosphorylcholine, ↓myo-inositol); αSyn species differentially alter amino acid metabolism: Glycine is increased by monomers/oligomers but decreased by fibrils, glutamine is reduced with fibrils, and alloisoleucine is decreased with monomers, indicating disrupted neurotransmission and metabolic adaptation; ↓Taurine and ↓dimethylamine with fibrils indicate oxidative and mitochondrial stress; ↑ATP suggests altered bioenergetics; Cholinergic impairment and excitatory–inhibitory neurotransmitter imbalance (↓ACh; altered glycine/taurine)
[46]	Primary mouse cortical neurons	WT recombinant human PFF; 1 µM; 5–14 dpt		• Energy metabolism	• ATP	Accumulation of misfolded proteins within mitochondria caused significant ATP depletion

Abbreviations used: *ACh* acetylcholine, *ATP* adenosine triphosphate, *AMPK *AMP-activated protein kinase, *ClpP* Caseinolytic mitochondrial matrix peptidase, *Cyt* *C* Cytochrome C, *Dpt* day post treatment, *hiPSC 3GPC* sn-3-glycero-phosphocholine human induced pluripotent stem cells, *MeSH* medical subject headings, *mPTP* mitochondrial permeability transition pore, *NAD+* nicotinamide adenine dinucleotide, *NADH* nicotinamide adenine dinucleotide phosphate, *NBDG* 2-(N-(7-nitrobenz-2-oxa-1,3-diazol-4-yl)amino)−2-deoxyglucose, *ns* not stated, *NMR* nuclear magnetic resonance, *PARP* Poly(ADP-ribose) polymerase, *PC* phosphorylcholine, *PFF* preformed fibrils *Ref* reference, *WT* wild type

### Synthesis of Findings

#### Mitochondrial Membrane Potential and Mitochondrial Respiration

Changes in mitochondrial membrane potential were measured in 21 studies, with membrane depolarisation most frequently reported following αSyn exposure [39–46]. Impaired oxidative phosphorylation, ATP production and mitochondrial respiration were also identified following exposure to oligomeric or fibrillar αSyn [42, 47−59]. More specifically, reductions in basal and maximal respiration, ATP depletion and altered bioenergetic profiles were common features. In some models, mitochondrial function was improved by protective interventions, including SS-31, a small synthetic aromatic-cationic tetrapeptide from the Szeto–Schiller peptide family that selectively targets the inner mitochondrial membrane and protects against oxidative stress [47], and Poly(ADP-ribose) polymerase −1 (PARP-1) inhibition [60].

#### Oxidative Stress and ROS Generation

Evidence of oxidative stress was a common finding [41, 48, 61–63]. Elevated ROS was found across 12 studies which assessed this outcome [21, 39, 42, 43, 46, 49, 52, 53, 55, 58, 64−66]. Sources of oxidative stress included impaired electron transport, NADPH oxidase activation, and metal-dependent redox interactions. ROS production was closely linked to lipid peroxidation, protein oxidation, and apoptotic signaling.

#### Mitochondrial Dynamics, Protein Import and Morphology

Only 10 of these studies investigated structural effects of misfolded αSyn stress on mitochondria. These studies demonstrated structural impairment of mitochondria, including fragmentation, disrupted cristae, and dysregulation of fission/fusion proteins [47, 49–51, 55, 67−69]. Oligomeric αSyn bound to TOM20 and disrupted mitochondrial protein import [45, 48, 58], linking aggregation directly to respiratory failure.

#### Mitochondrial Quality Control Including Mitophagy

Evidence of altered mitophagy was reported in 3 studies out of 5 studies assessed this outcome [45, 50, 65, 67, 68]. Although Parkin recruitment was observed, clearance of damaged mitochondria was often incomplete, leading to accumulation of dysfunctional organelles. Crosstalk with tau pathology [67] further implicated αSyn in broader proteostatic stress.

#### Non-neuronal Cell Vulnerability

αSyn-induced mitochondrial dysfunction was also observed in several non-neuronal cell types, including astrocytes [40, 44], microglia [42, 43, 48], endothelial cells [41] and fibroblasts [59]. Importantly, the mitochondrial phenotype and its downstream consequences differed according to cell type. In astrocytes, αSyn aggregates impaired mitochondrial respiration and bioenergetic capacity, with greater vulnerability observed in PARK2-variant cells [44]. Similar findings indicated that astrocytic mitochondrial dysfunction occurred downstream of membrane rigidity, endoplasmic reticulum stress and calcium dysregulation [40]. Together, these findings suggest that αSyn exposure can compromise the metabolic and homeostatic functions of astrocytes.

In microglia, mitochondrial dysfunction was more closely associated with inflammatory signaling. αSyn-induced mitochondrial oxidative stress acted upstream of endoplasmic reticulum stress and TXNIP/NLRP3 inflammasome activation, promoting a pro-inflammatory phenotype [42]. αSyn-dependent NOX2 activation provided an additional source of oxidative stress in microglial models [43, 48], indicating that mitochondrial and inflammatory responses may be closely interconnected in these cells. In endothelial cells, αSyn-associated mitochondrial dysfunction was accompanied by impaired barrier integrity and was modified by interactions with astrocytes and microglia [41], suggesting a potential link between mitochondrial stress and neurovascular dysfunction. Fibroblasts also exhibited impaired mitochondrial respiration following αSyn exposure [59], demonstrating that these mitochondrial effects were not restricted to neural cell types.

Collectively, these findings indicate that αSyn-associated mitochondrial dysfunction is not a uniform cellular response. Rather, its functional consequences appear to depend on cellular context, ranging from impaired bioenergetic and homeostatic support in astrocytes, to inflammatory activation in microglia and compromised barrier function in endothelial cells. This cellular heterogeneity highlights the importance of considering both neuronal and non-neuronal responses when evaluating the mitochondrial consequences of αSyn pathology.

#### Genetic and Molecular Modifiers

Several studies investigated whether PD-associated genetic backgrounds or αSyn variants modified susceptibility to αSyn-induced mitochondrial dysfunction. Compared with wild-type αSyn, treatment of cells with the A53T variant was associated with greater mitochondrial membrane disruption, nitric oxide release and S-nitrosylation, impaired mitochondrial quality control and increased apoptosis [61]. Treatment with A53T αSyn was also associated with enhanced cardiolipin binding and aggregation, alongside impaired complex I activity, increased mitochondrial ROS production, mitochondrial membrane permeabilisation and cell death [52]. Treatment of cells with the A30P variant also demonstrated cardiolipin binding, although to a lesser extent than A53T [52], while exposure to the A30P/A53T double mutant resulted in more pronounced mitochondrial dysfunction than wild-type αSyn [40]. Similarly, treatment with the E46K variant produced greater mitochondrial impairment than wild-type αSyn, characterised by reduced mitochondrial activity, increased ROS production, prolonged aggregate–membrane interactions and oxidative damage [66]. Genetic backgrounds associated with PD also influenced cellular vulnerability to exogenous αSyn. PARK2-variant hiPSC-derived astrocytes exhibited a greater reduction in basal and coupled mitochondrial respiration following αSyn fibril exposure than control astrocytes [44]. Neurons carrying SNCA triplication similarly showed increased susceptibility to αSyn aggregate-induced toxicity, including greater mitochondrial permeability transition pore formation, increased cytosolic and mitochondrial ROS generation, glutathione depletion, lipid peroxidation and neuronal cell death [21, 55]. Collectively, these findings indicate that disease-associated αSyn variants and the specific PD-associated genetic backgrounds examined can amplify cellular vulnerability to αSyn-mediated mitochondrial stress.

Several additional molecular pathways were identified as modifiers of the mitochondrial response to αSyn, including p-NMDAR2B/Nur77 suppression [49], P2X7 receptor activation [53, 56] and PARP-1 overactivation [60]. Suppression of p-NMDAR2B/Nur77 signalling disrupts calcium-dependent survival pathways and promotes mitochondrial depolarisation [49]. P2X7 receptor activation contributes to calcium dysregulation, ROS production and inflammatory signalling [53, 56], while PARP-1 overactivation following oxidative DNA damage promotes NAD⁺ and ATP depletion, contributing to bioenergetic failure and neuronal death [60].

#### Source of αSyn Monomers and Oligomer/Fibril Production Protocols

Included studies used recombinant αSyn as the starting material for the generation of the exogenously applied αSyn species; none used patient-derived, amplified patient material or brain homogenate-derived αSyn. For PFF generation, the most commonly reported approach involved incubation at 37 °C with continuous agitation, typically for approximately 7 days at 1000 rpm [48, 50, 54, 60, 63]. However, substantial variation was evident between studies, with reported shaking speeds ranging from approximately 600 to 1400 rpm [40, 43] and incubation periods ranging from 5 days to 2 weeks [39, 42, 57, 67, 69]. Sonication of the resulting fibrils prior to cellular exposure was also commonly employed, although this was not universal [49, 62]. One study specifically avoided sonication on the basis that fragmentation of fibrils into smaller species may reduce their resemblance to aggregates present in the PD brain [40]. Further methodological variation included the use of different buffer conditions to generate distinct αSyn fibrillar polymorphs, including fibrils, ribbons, F65 and F91 species [44]. One study used protein misfolding cyclic amplification (PMCA) rather than conventional prolonged fibril formation, generating fibrillar αSyn through repeated cycles of sonication and agitation [70]. Protocols used to generate oligomeric αSyn showed even greater methodological variation. Most preparations involved incubation at 37 °C, but preparation times ranged from several hours to 3 days and agitation speeds ranged from approximately 100 to 1400 rpm [21, 41, 47, 52, 55, 57, 58, 64, 65]. Some protocols generated oligomers without agitation [59], while others used relatively high agitation over substantially shorter preparation periods; for example, 1400 rpm for 30 min [51]. A further study used a distinct cold-induction protocol, incubating αSyn at 4 °C for 6 h with agitation at 100 rpm [61]. Collectively, these studies demonstrate that, although recombinant αSyn was consistently used as the starting material, substantial variation existed in the conditions used to generate oligomeric and fibrillar species, particularly with respect to incubation temperature and duration, agitation, sonication and fibril polymorphism.

#### Summary of Results

The biological effects of exogenously applied αSyn differed according to its conformational state. Monomeric αSyn generally produced minimal or no measurable impairment of mitochondrial integrity or function when compared with aggregated species [21, 40, 44, 50, 52, 54, 55, 58, 70]. In contrast, oligomeric αSyn was more consistently associated with mitochondrial membrane depolarisation, increased ROS production, impaired respiration and ATP production, and activation of apoptotic pathways [21, 47, 51–53, 55, 58, 59, 61, 64–66]. These effects were not uniform across experimental systems; for example, acute oligomer exposure did not impair global respiration in mixed neuronal cultures [51], highlighting the influence of concentration, exposure duration and cell type.

Fibrillar αSyn and PFFs were similarly associated with mitochondrial dysfunction, but were more frequently linked to broader structural and mitochondrial quality-control abnormalities. Across the included studies, fibrillar species impaired mitochondrial respiration and bioenergetic capacity, increased oxidative stress, disrupted mitochondrial protein import, altered mitochondrial morphology and promoted defective mitophagy [39, 42, 46, 48, 50, 54, 60, 62, 63, 67, 70]. However, responses again varied between models; notably, PMCA-generated fibrillar αSyn produced increased mitochondrial respiration rather than immediate respiratory impairment [70]. A small number of studies also examined distinct fibrillar preparations or polymorphs, although the available evidence was insufficient to determine whether individual fibrillar polymorphs produced reproducibly distinct mitochondrial phenotypes.

Genetic background and disease-associated αSyn variants also modified the severity of these effects. A53T, A30P/A53T and E46K αSyn variants were associated with greater mitochondrial impairment than wild-type αSyn, while PARK2-variant astrocytes and neurons carrying SNCA triplication demonstrated increased vulnerability to exogenous αSyn exposure [21, 40, 44, 52, 55, 61, 66]. These findings suggest that the mitochondrial response to αSyn is influenced not only by protein conformation, but also by the underlying genetic context of the model.

Overall, the 33 included studies support mitochondrial dysfunction as a recurring consequence of exogenous αSyn exposure across diverse in vitro systems. Aggregated αSyn species, particularly oligomeric and fibrillar forms, were more consistently associated with mitochondrial impairment than monomeric αSyn, with common outcomes including membrane depolarisation, impaired bioenergetics, increased oxidative stress, disrupted mitochondrial structure and defective quality control pathways. However, the magnitude and nature of these effects varied according to αSyn conformation and preparation, concentration, exposure duration, cellular model and genetic background. Collectively, this convergence across multiple experimental systems supports mitochondrial dysfunction as a central feature of αSyn-mediated cellular pathology, while also highlighting the methodological and biological heterogeneity that complicates direct comparison between studies.

## Discussion

Mitochondrial dysfunction has been widely recognised as having a role the pathogenesis of synucleinopathies [71] and understanding how αSyn species affect mitochondrial function leading to cellular degeneration may lead to a better understanding of disease mechanisms and ultimately novel therapeutics. The key finding of this review is that across cell model systems, mitochondrial function is impaired following exposure to oligomeric and fibrillar αSyn species. Cellular phenotypes showing mitochondrial membrane depolarisation, complex-1 dysfunction, increased reactive oxygen species and impaired mitophagy were reported, supporting the view the mitochondrial dysfunction represents a convergent mechanism through which pathological αSyn species could contribute to cellular toxicity. However, these effects were not uniform across cell models with evidence of different components of mitochondrial function impaired in response to αSyn, likely due to heterogeneity in experimental conditions. Instead, αSyn-induced mitochondrial dysfunction appeared to vary according to αSyn conformation, exposure duration, concentration, cell type, genetic background or with outcome measures selected. This review highlights the potential utility of cellular models to study the effects of αSyn on mitochondrial function as an early process in the pathology of synucleinopathies.

Changes in brain metabolism have been described in individuals with synucleinopathy-related disease. FDG-PET studies in PD, PDD, DLB and prodromal αSyn-related disease have reported regional hypometabolism, particularly in cortical and posterior brain regions, with some evidence that metabolic change may preclude structural atrophy during cognitive decline [72–74]. However, these changes in brain energy metabolism could be linked to synaptic dysfunction, neuronal loss, altered network activity, glial responses and vascular factors. To understand the specific mechanistic pathways underlying altered brain energy metabolism ‘in vitro’ models of alpha synuclein pathology are critical. In this review, mitochondrial effects were widespread including complex-I impairment, NADH accumulation, mitochondrial membrane potential disruption and activation of apoptotic pathways supporting an association between impaired oxidative phosphorylation and cell death pathways (see Table 1). These findings suggest that disease-associated αSyn species can reproduce, at the cellular level, several features of mitochondrial impairment relevant to synucleinopathies.

Oxidative stress is commonly implicated in the pathology of PD and related synucleinopathies, which provides a mechanism linking αSyn and mitochondrial dysfunction [75]. However, whether oxidative stress drives the disease process or appears as a function of mitochondrial dysfunction is less clear. Therefore, investigating alterations in oxidative stress ‘in vitro’ can provide mechanistic insight into whether αSyn exposure directly induces redox imbalance alongside mitochondrial dysfunction. In fact, oxidative stress was one of the most frequently reported outcomes across the included studies. Increased reactive oxygen species generation was observed across several oligomeric and fibrillar αSyn models, often alongside mitochondrial depolarisation, lipid peroxidation, antioxidant depletion and impaired respiratory capacity [39, 49, 53, 55]. Neuronal cells are particularly susceptible to oxidative stress in the brain due to high oxygen consumption, lipid-rich membranes and high calcium traffic across membranes [76]. However, in this review both microglial and astrocyte models also demonstrated elevated oxidative stress more closely linked to inflammatory processes [42–44, 48, 62], suggesting that αSyn-associated oxidative stress generates a redox-active environment in which mitochondrial dysfunction, inflammatory activation and impaired antioxidant support interact to amplify vulnerability across cell types. This highlights a limitation in current models investigating αSyn exposure, where the interaction between different cell types is lost. For example, mitochondrial dysfunction in microglia may contribute to neurodegeneration indirectly by shaping inflammatory and redox environments. Studies using astrocytes also showed impaired respiration, delayed mitochondrial depolarisation, ER stress and calcium-associated dysfunction following αSyn exposure [41, 44]. As astrocytes provide neuronal metabolic and neurotransmission support, astrocytic mitochondrial dysfunction may weaken the cellular support systems that normally protect neurons. These observations argue strongly for greater use of neuron–glia co-cultures and multicellular systems. While targeting mitochondria using SS-31 drugs showed promising results in in vitro models, these results may not necessarily translate into in vivo models, as they lack vascular, lymphatic clearance, and immune microenvironment, limiting their translational value. Genetic background further modified mitochondrial vulnerability in reviewed studies. Models involving A53T, E46K, SNCA triplication or PARK2-associated systems often showed amplified mitochondrial impairment, including altered membrane potential, increased ROS, impaired respiration or reduced metabolic resilience [51, 53, 60, 61]. These findings are valuable because they suggest that exogenous αSyn toxicity interacts with pre-existing genetic susceptibility. However, they also complicate interpretation. In genetically vulnerable models, mitochondrial dysfunction may reflect the combined effects of αSyn exposure, baseline proteostatic stress and inherited mitochondrial susceptibility. Future studies should therefore include appropriate isogenic controls and distinguish between αSyn-induced effects and background vulnerability.

Evidence from the identified papers also highlighted that αSyn exposure can disrupt mitochondrial structure with fragmentation, swelling and altered mitophagy leading to the accumulation of damaged mitochondria [7, 45, 49, 63]. This suggests mitochondrial function is altered through structural damage in addition to bioenergetic stress. αSyn species have been shown to interfere with mitochondrial protein import through TOM20-related pathways, disrupt mitochondrial-associated chaperones such as Hsp60, impair mitochondrial proteostasis and alter Parkin-associated mitophagy [6, 45, 62, 77]. These mechanisms are particularly important because neurons rely on mitochondrial quality control to maintain synapses and axonal projections. If damaged mitochondria are not efficiently repaired, trafficked or removed, they may become chronic sources of reactive oxygen species, calcium dysregulation and apoptotic signaling. In addition to functional and structural damage to mitochondria, metabolite concentrations were also altered in cellular models of αSyn exposure. αSyn exposure was associated with altered glucose uptake and reduced ATP levels in neurons. In hiPSC-derived microglia, αSyn exposure was also associated with a shift towards glycolysis, consistent with inflammatory activation and mitochondrial stress [49, 51, 54, 55]. This suggests changes in metabolic pathways precede cell death and may be an early indicator of αSyn toxicity. Future studies comprehensively profiling metabolite concentrations in response to αSyn exposure may highlight pathways most affected and aid in identification of future therapeutic targets.

The reviewed in vitro findings are broadly consistent with evidence from human post-mortem PD brain tissue, supporting the translational relevance of the mitochondrial phenotypes identified across the included cellular models. Reduced mitochondrial complex I activity [78–80], increased oxidative damage [81] and lipid peroxidation [82, 83], respiratory-chain deficiencies, and accumulation of mtDNA deletions [84–86] have all been reported in PD brain tissue, particularly within the substantia nigra. These observations parallel the impaired oxidative phosphorylation, increased ROS production and bioenergetic dysfunction observed following αSyn exposure in the in vitro studies included in this review. Post-mortem studies have also demonstrated microgliosis [87, 88], elevated inflammatory markers [42, 89, 90] and astrocytic abnormalities [40, 91], consistent with our observation that αSyn-associated mitochondrial dysfunction extends beyond neurons and may interact with inflammatory and glial responses.

Importantly, several mechanistic observations made in vitro also have direct parallels in human tissue. Aberrant αSyn–TOM20 interactions have been identified in nigrostriatal dopaminergic neurons from PD brains and associated with impaired mitochondrial protein import [58], supporting a mechanism also identified across the cellular models reviewed here. Similarly, phosphorylated αSyn aggregates have been observed in association with mitochondrial damage, fragmentation and altered mitophagy in post-mortem tissue [45]. Together, these findings suggest convergence between experimental models and human PD pathology across mitochondrial bioenergetics, oxidative stress, protein import and mitochondrial quality-control pathways. However, post-mortem tissue predominantly reflects advanced or terminal disease and cannot establish the temporal sequence of these abnormalities or determine whether mitochondrial dysfunction is an initiating event, a consequence of progressive neurodegeneration, or part of a reciprocal pathological process. In vitro models therefore remain valuable for isolating the direct effects of defined αSyn species and investigating mitochondrial changes that may occur before overt cell loss.

### Limitations and Future Directions

Several considerations should be taken into account when interpreting the findings of this review. First, the review was intentionally restricted to in vitro studies in which αSyn was applied exogenously as monomeric, oligomeric or fibrillar species. Models based primarily on endogenous αSyn overexpression or genetically driven intracellular accumulation were therefore outside the predefined inclusion criteria. This focused approach enabled direct comparison of mitochondrial responses to defined exogenous αSyn species, but it also means that the findings do not capture the full range of cellular models used to investigate αSyn pathology. Future reviews and experimental studies could address this by directly comparing exogenous αSyn models with overexpression- and mutation-based systems to determine whether common mitochondrial mechanisms emerge across different approaches to modelling αSyn pathology.

Second, substantial methodological heterogeneity was evident across the included studies. Studies differed in αSyn species, preparation method, dose, sonication protocols, aggregation state, exposure duration, cellular model and mitochondrial outcome measures. Importantly, oligomers, protofibrils, mature fibrils and sonicated PFFs represent related but biologically distinct αSyn species, with differences in their size, structure, cellular uptake, seeding efficiency and toxicity [20]. In addition, three of the 33 included studies did not report either αSyn dosage [46] or processing time [52, 55], further limiting reproducibility and direct comparison between studies. Greater standardisation of αSyn preparation and characterisation, together with more consistent reporting of experimental conditions, would improve comparability across future studies. Future work would also benefit from incorporating complementary measures of mitochondrial function, including bioenergetics, membrane potential, oxidative stress, morphology and mitochondrial quality-control pathways, rather than relying on individual endpoints.

Finally, the translational relevance of mitochondrial protection observed in vitro should be considered within the context of model complexity. Mitochondrial-targeted interventions, including SS-31, demonstrated protective effects in some of the cellular models reviewed; however, isolated in vitro systems cannot fully reproduce the integrated vascular, immune and clearance systems present within the human brain, or the complex interactions between neuronal and non-neuronal cell populations. Rather than diminishing the value of these models, this highlights their utility as mechanistic platforms for identifying candidate pathways that can subsequently be tested in increasingly physiologically relevant systems. Immortalised cell lines remain valuable for mechanistic investigation but cannot fully model neuronal maturity, cell–cell communication or disease-specific vulnerability. Human iPSC-derived neurons, astrocytes and microglia, particularly when combined with isogenic controls and disease-associated genotypes, provide greater human relevance. Future studies incorporating neuron–glia co-cultures, multicellular iPSC-derived systems, organoids and complementary in vivo approaches may therefore help establish which αSyn-associated mitochondrial mechanisms are maintained across increasing levels of biological complexity. Such stepwise validation will be important for determining whether mitochondrial pathways identified in cellular models represent viable therapeutic targets in PD and related synucleinopathies.

## Conclusion

This systematic review identifies mitochondrial dysfunction as a convergent mechanism underlying αSyn-mediated neurodegeneration. The evidence positions mitochondria as active participants in αSyn pathology, translating proteostatic stress into cellular dysfunction through energy failure, redox disruption and impaired mitochondrial quality control. Across mammalian in vitro models, exogenous αSyn species, particularly oligomeric and fibrillar forms, consistently impair mitochondrial bioenergetics, disrupt oxidative phosphorylation, and compromise organelle quality control. These alterations collectively generate oxidative stress, bioenergetic insufficiency, and defective mitophagy that precedes and potentiate neuronal injury. The reciprocal interaction between αSyn aggregation, mitochondrial dysfunction, and impaired organelle turnover forms a pathogenic cycle that accelerates synaptic and neuronal degeneration characteristic of synucleinopathies. These findings identify mitochondrial dysfunction as a potential therapeutic target in PD and related synucleinopathies. However, the protective effects observed in in vitro models require validation in more complex multicellular, in vivo and human systems before mitochondrial-targeted approaches can be considered viable disease-modifying therapies. Continued research into the development of human disease-relevant models of synucleinopathies could facilitate future therapeutic discovers and enable larger scale, early testing.

## Data Availability

No datasets were generated or analysed during the current study.

## Abbreviations

*ATP*: Adenosine triphosphate
*MeSH*: Medical subject headings
*ΔΨm*: Mitochondrial membrane potential
*mPTP*: Mitochondrial permeability transition pore
*NADH*: Nicotinamide adenine dinucleotide phosphate
*PARP-1*: Poly(ADP-ribose) polymerase-1
*ROS*: Reactive oxygen species
